# PHYSICAL RESERVE MEASURES AS PREDICTORS OF RESILIENCE IN OLDER ADULTS UNDERGOING TOTAL KNEE REPLACEMENT

**DOI:** 10.1093/geroni/igad104.0377

**Published:** 2023-12-21

**Authors:** Maria Sison, Richard Sloane, Cathleen Colon-Emeric, Heather Whitson

**Affiliations:** Duke University, Durham, North Carolina, United States; Duke University Medical Center, Durham, North Carolina, United States; Duke University, Durham, North Carolina, United States; Duke University, Durham, North Carolina, United States

## Abstract

Measures of physical reserve have been shown to predict resilience following surgery in older adults. The objective of this study was to evaluate the utility of gait speed, hand grip strength, and 3-minute walk distance to predict resilience in older adults scheduled for total knee arthroplasty (TKA), in whom preoperative gait and mobility may be limited by pain or reduced range of motion. We conducted an interim analysis of data from PRIME-KNEE, an ongoing prospective study following participants age ≥60 years undergoing TKA recruited from Duke hospitals. We determined the change in self-reported Lower Extremity Physical Activities of Daily Living (LE PADL) score from pre-surgery baseline to 4 months following TKA in 133 participants (mean age = 72, 59% female). Gait speed, hand grip, and 3-minute walk were collected at baseline. A total of 110 (83%) participants exhibited “resilience,” defined dichotomously as any improvement in LE PADL score (occurred in 38 participants) or return to a non-disabled state (occurred in 72 participants). Logistic regression analysis adjusted for age and sex revealed that 3-minute walk distance was significantly associated with resilience at 4 months (OR=1.012 [CI 1.002-1.022]) with an area under the curve of 0.634. The presentation will review the implications of these results and present findings with an updated sample, as well as discuss the need for other predictive measures, such as provocative tests (i.e., tests that measure response to an experimental stressor) and biomarkers.

